# Non-ischemic cardiomyopathy after rituximab treatment for membranous nephropathy

**DOI:** 10.15171/jrip.2017.04

**Published:** 2016-11-02

**Authors:** Wisit Cheungpasitporn, Stephen L. Kopecky, Ulrich Specks, Kharmen Bharucha, Fernando C. Fervenza

**Affiliations:** ^1^Division of Nephrology and Hypertension, Department of Internal Medicine, Mayo Clinic, Rochester, MN, USA; ^2^Department of Cardiology, Mayo Clinic, Rochester, MN, USA; ^3^Division of Pulmonary and Critical Care Medicine, Department of Internal Medicine, Mayo Clinic, Rochester, MN, USA

**Keywords:** Rituximab, Membranous nephropathy, Cardiovascular disease, Cardiomyopathy, Adverse effect

## Abstract

Rituximab is an anti-CD20 monoclonal antibody frequently used for the treatment of non-Hodgkin’s lymphoma, chronic lymphocytic leukemia (CLL), rheumatoid arthritis (RA), and anti-neutrophilic cytoplasmic antibody (ANCA)-associated vasculitis. In addition, rituximab has recently been increasingly used as an off-label treatment in a number of inflammatory and systemic autoimmune diseases. It is advised that rituximab infusion may cause infusion reactions and adverse cardiac effects including arrhythmia and angina, especially in patients with prior history of cardiovascular diseases. However, its detailed cardiotoxicity profile and effects on cardiac function were not well described. We report a 51-year-old man who developed non-ischemic cardiomyopathy after rituximab treatment for membranous nephropathy. The patient experienced reduced cardiac functions within 48 hours after the initial infusion, which remained markedly reduced at 9-month follow-up. As the utility of rituximab expands, physicians must be aware of this serious cardiovascular adverse effect.

**Implication for health policy/practice/research/medical education:**

Rituximab is currently approved by the US Food and Drug Administration (FDA) for the treatment of non-Hodgkin’s lymphoma (NHL), chronic lymphocytic leukemia (CLL), rheumatoid arthritis (RA), antineutrophil cytoplasmic antibody (ANCA)-associated vasculitis. In addition, rituximab has recently been used as an off-label treatment in a number of diseases. However, its detailed cardiotoxicity profile and effects on cardiac function are not clearly described in the literature. This case of non-ischemic cardiomyopathy after infusion of rituximab for membranous nephropathy highlights the growing evidence that rituximab can affect cardiac function and requires greater attention as a causative agent of cardiomyopathy.

## Introduction

Rituximab, a chimeric monoclonal anti-CD20 antibody, is currently approved by the US Food and Drug Administration (FDA) for the treatment of non-Hodgkin’s lymphoma (NHL), chronic lymphocytic leukemia (CLL), rheumatoid arthritis (RA), antineutrophil cytoplasmic antibody (ANCA)-associated vasculitis including granulomatosis with polyangiitis (GPA) and microscopic polyangiitis (MPA) (1-5). In addition to these indications, the use of rituximab has been expanded as an off-label treatment for many diseases including systemic lupus erythematosus, Sjögren’s syndrome, idiopathic thrombocytopenic purpura, bullous dermatologic diseases, membranous nephropathy, steroid-dependent or frequently relapsing idiopathic nephrotic syndrome, treatment in recurrent and de novo glomerular disease after renal transplantation, and others (6-9).

Despite its relative safety profile, infusion-related side effects of rituximab, such as fevers, chills and rigors are common, reported to be as high as 87% (2-5,10,11). Due to reported cases of angina, acute coronary syndrome (ACS), and arrhythmias related to rituximab infusion, caution for its use is advised by FDA in patients with a history of cardiovascular disease (12). However, its detailed cardiotoxicity profile and effects on cardiac function are not clearly described in the medical literature. We report a case of a 51-year-old man who developed non-ischemic cardiomyopathy after receiving his first infusion of rituximab for membranous nephropathy. This case highlights the growing evidence that rituximab can affect cardiac function and requires greater attention as a potential causative agent of cardiomyopathy.

## Case Presentation

A 51-year-old man was diagnosed with phospholipase A2 receptor (PLA2R) positive, primary membranous nephropathy. He was initially treated with prednisone and cyclophosphamide without significant improvement. The patient was subsequently started on tacrolimus and became calcineurin inhibitor-dependent. Due to a relapse despite being on tacrolimus, rituximab treatment was recommended. His past medical history was remarkable for multiple episodes of deep venous thrombosis on chronic anticoagulation, hypertension, and dyslipidemia. He did not have any previous cardiac history and denied any concerning preceding cardiac symptoms of chest pain, dyspnea, syncope, orthopnea, or paroxysmal nocturnal dyspnea. The patient had a 33-pack-year history of smoking but quit two years previously. His 12-lead electrocardiogram (ECG) prior to rituximab treatment showed normal sinus rhythm. He worked as a carpenter and had been carrying heavy equipment at work without significant physical limitation. His family history is significant for coronary artery disease in his father at 50 years of age and dilated cardiomyopathy (DCM) in his brother. Due to significant cardiovascular diseases in his family members the patient underwent a cardiolite treadmill stress test which showed normal exercise tolerance and functional class I on Bruce protocol for 15 minutes. There was no evidence of fixed or reversible defect.

The patient was 179 cm tall with a body weight of 88 kg and body surface area (BSA) of 2.1 m^2^. Rituximab was administered at a dosage of 1000 mg intravenous (IV) at a starting infusion rate of 50 mL/h. Oral acetaminophen (1000 mg), oral diphenhydramine (50 mg), and IV methylprednisolone (100 mg) were also given as premedication. He underwent his first rituximab infusion without any immediate side effects. Forty-eight hours after the infusion, the patient presented to the hospital and reported having woken up from sleep with chest tightness and shortness of breath. He also had nausea and emesis. His physical examination was unremarkable with no evidence of heart failure.

**Figure 1:**
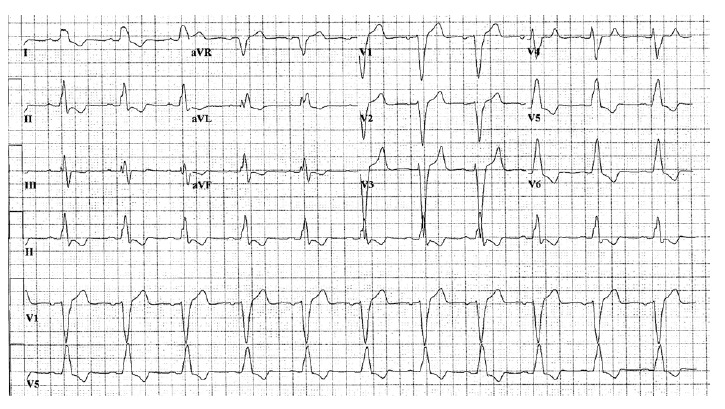
A 12-lead electrocardiogram showed ventricular rate of 68 beats/min, normal sinus rhythm, and left bundle branch block.

A 12-lead ECG showed a new left bundle branch block (LBBB) as shown in Figure 1. Troponin I level was 0.08 ng/mL (normal <0.03 ng/mL). Otherwise, Laboratory testing revealed the following values: hemoglobin 11.2 g/dL (reference range 13.5-17.5 g/dL), white blood cell count 9.6×10^9^/L (reference range 3.5-10.5 ×10^9^/L), platelet 157×10^9^/L (reference range 150-450 ×10^9^/L), serum creatinine 1.5 mg/dL (reference range 0.8-1.3 mg/dL), BUN 41 mg/dL (reference range 8-24 mg/dL), sodium 141 mmol/L (reference range 135-145 mmol/L), potassium 5.0 mmol/L (reference range 3.6-5.2 mmol/L), chloride 111 mmol/L (reference range 100-108 mmol/L), bicarbonate 22 mmol/L (reference range 22-29 mmol/L). Computed tomography angiogram of the chest was negative for pulmonary embolism without evidence of significant coronary calcification. An urgent transthoracic echocardiogram demonstrated moderately reduced left ventricular (LV) systolic function with an ejection fraction (EF) of 30% with moderate hypokinesis of the anteroseptal myocardium. There was no significant valvular disease. The patient subsequently underwent left and right heart catheterization with coronary angiography which revealed a cardiac output of 5.9 L/min (normal range: 4-8L/min) and a cardiac index of 2.88 L/min/m^2^(normal range: 2.6–4.2 L/min/m^2^). There was no significant atherosclerotic disease noted in the coronary vessels. However, there was moderate diffuse hypokinesis of the LV on ventriculogram. Right heart catheterization demonstrated mildly elevated right and left-sided filling pressures. Thus, the diagnosis of non-ischemic cardiomyopathy was made, and the underlying etiology of his non-ischemic cardiomyopathy was postulated to be due to rituximab treatment.

The patient received medical treatment for the cardiomyopathy with carvedilol 3.125 mg orally twice a day as well as lisinopril 10 mg and rosuvastatin 10 mg orally once a day. The patient was also subsequently placed on a life vest due to prevent sudden cardiac death. The patient was restarted on tacrolimus for his treatment of membranous nephropathy. He did not receive any further treatment with rituximab. A repeat echocardiogram one month later still demonstrated severe LV enlargement with a calculated EF of 26%. There was persistent generalized LV hypokinesis with abnormal ventricular septal wall motion due to LBBB. Otherwise, there was a mild valvular heart disease. His follow-up ECG showed normal sinus rhythm with LBBB. Carvedilol was increased to 6.25 mg twice a day and slowly titrated up to 25 mg twice a day. At 9-month follow-up, despite that he had better optimization of his medical therapy, his transthoracic echocardiogram still showed reduced LVEF of 31% with moderate−severe LV enlargement. Thus, the patient was evaluated and underwent cardiac resynchronization therapy with implantable cardioverter defibrillators (CRT-D) implantation. At 3 month after initiation of CRT therapy, the patient had significant improvement in his functional status with LVEF of 52%. Although the patient still had proteinuria at 607 mg/dL/24 hours, his kidney function remained stable with creatinine of 1.6 mg/dL and creatinine clearance of 69 mL/min/BSA.

## Discussion

Rituximab, B-cell-depleting agent targeting CD20 antigen, is being increasingly and effectively used “off label” for various conditions including glomerular diseases such as lupus nephritis, membranous nephropathy, focal segmental glomerulosclerosis, cryoglobulinemic glomerulonephritis, antibody-mediated renal allograft rejection and recurrent glomerulonephritis in renal allograft (6-9,13). However, the data regarding its cardiac side effect profile are not well recognized. We report a rare, but serious cardiac adverse effect of irreversible/partial reversible non-ischemic cardiomyopathy following rituximab treatment in a patient with membranous nephropathy.

To identify potential previously reported cases of potential cardiovascular adverse effects related to rituximab treatment, the literature searches of Embase, Medline and Cochrane through July 2016 was performed using the terms “rituximab” and “rituxan” combined with the terms “cardiac”, “cardiovascular”, “cardiomyopathy”, “coronary” and “heart”. Not surprisingly, some reports have stated that rituximab is safe for the heart (2-5). This finding is also consistent with the data from our recent published data from a multicenter, randomized, double-blind controlled trial comparing the efficacy of rituximab with conventional immunosuppression with cyclophosphamide for the treatment of severe ANCA–associated vasculitis in 197 patients (1). There was no significant difference in serious or non–disease-related adverse event between two groups. Also, significant or serious cardiovascular adverse effects were not noted.

Rituximab is well known to cause infusion-related side effects from cytokine release, such as fevers and rigors within the ﬁrst few hours, especially during the ﬁrst infusion. These symptoms are associated with cytokine release, particularly interleukin 6 (IL-6). Most of these cases resolve promptly with supportive management. Hypotension, angioedema, hypoxia, and bronchospasm can also be seen in up to 10% of cases (10,11). With previously reported cases of angina, ACS, and arrhythmias with rituximab infusion (14-25), FDA has cautiously provided recommendations to discontinue infusions for serious or life-threatening cardiac arrhythmias and perform cardiac monitoring during and after all infusions of rituximab for patients who develop clinically significant arrhythmias, or who have a history of arrhythmia or angina (12). However, these current recommendations do not sufficiently address the cardiovascular concerns related to rituximab treatment, since cases with adverse cardiovascular effects following rituximab infusion have been additionally reported as shown in Table 1. In addition to ACS and arrhythmia, rituximab-induced cardiogenic shock, delayed reduction in LVEF, Takotsubo’s and non-ischemic cardiomyopathies have also been described (14-23,25,26) as summarized in Table 2.

**Table 1:** Reported cases with cardiovascular adverse effect following rituximab infusion

	**Year**	**No. of cases**	**Demographics**	**Indication for Rituximab treatment**	**Cardiovascular toxicities**	**Onset**	**Prior cardiac history**	**Electrocardiogram (Post infusion)**	**Echocardiogram**	**Coronary angiography**	**Outcomes**
Garypidou et al‏(14)	‏2004	1	A 71-year-old male	Non-Hodgkin lymphomas and B-cell chronic lymphocytic leukemia	Unstable angina/acute coro-nary syndrome	Four hours after the initiation of the ﬁrst infusion	Myocardial infarction 12 years previously	Sinus tachycardia with no ST-segment or T-wave changes	NR	NR	The pain resolved with the administration of nitroglycerin sublingually and the infusion was interrupted. During the next few days, there was no elevation of the biochemical cardiac markers or ECG changes.
Millward et al‏(15)	‏2005	1	A 20-year-old female	Refractory thrombotic thrombocytopenia purpura	Cardiogenic shock	Six hours after the rituximab infusion	None	NR	Acute biventricular cardiogenic shock with EF of 5%-10%	NR	1 week after cardiogenic shock, biventricular systolic and diastolic functions were normalized.
Kanamori et al (16)	‏2006	3	A 80-year-old man, 65-year-old man, and 55 year-old man	Non-Hodgkin’s lymphoma	Delayed reduction in LV function	-Case 1: 2 weeks after rituxi-mab -Case 2: after the sixth course -Case 3: after the fourth course	-Case 1: no risk factor for cor-onary artery disease -Case 2: smoking -Case 3: smoking	NR	-Case 1: EF 35% 2 weeks after rituximab -Case 2: EF decreased to 50% after the sixth course from 65% before treatment -Case 3: EF decreased to 55% after the fourth course from 73% before treatment	NR	-Case 1: died -Case 2: asymptomatic -Case 3: recovered one month after treatment
Armitage et al (17)	‏2008	3	The median age of 61 years	Lymphoproliferative disorders	Acute coronary syndromes	Initial infusion of rituximab	One patient had known athero-sclerotic heart disease, and 2 patients had risk factors for coronary artery disease	NR	NR	NR	One patient died of an ar-rhythmia that deteriorated into asystole, and 2 patients recov-ered and underwent coronary angiography
Poterucha et al (18)	‏2010	‏1	A 79-year-old woman	Malignant lymphoma	Polymorphic ventricular tachycardia.	Thirty minutes into the initial infusion of rituximab	A history of atrial flutter post atrioventricular nodal ablation 1 year before, followed by placement of a pacemaker with 100% ventricular-paced	No ischemic changes, atrial fibrillation with VVIR pacing. The QT interval was technically prolonged. Interrogation of pacemaker’s intracardiac electrogram revealed a 12-second run of polymorphic VT	NR	NR	Discharged from the hospital a day later No recurrence of arrhythmia at 3-year follow-up
Lee et al (19)	‏2012	‏1	A 52-year-old woman	Follicular lymphoma	Coronary vasospasm	Within 10 minutes of first treatment	No cardiac risk factors other than a seven pack-year smoking history	New onset T-wave inversion in the anterior precordial leads	NR	Normal cardiac function with no evidence of occlusive disease in the coronary arteries	Rechallenged with rituximab with continuous cardiac monitoring. No recurrent chest pain or ECG changes
Passalia et al (20)	‏2013	‏2	A 75- year-old Caucasian man (case 1) and a 57- year-old Caucasian woman (case 2)	Stage IV non-Hodgkin lymphoma	-Case 1: New onset atrial fibrillation -Case 2: chest pain	-Case 1: Immediately after the end of the first infusion -Case 2: after the first 90 minutes of infusion	-Case 1 had a right bundle branch block and several cardiovascular risk factors: cigarette smoking, dyslipidemia, hypertension -Case 2 the patient was a smoker and had previous chemotherapy including high doses of anthracyclines (340 mg cumulative dose).	-Case 1: atrial fibrillation and confirmed RBBB. -Case 2: sinus tachycardia at a rate of 118 bpm.	-Case 1: normal LV function with an estimated EF of 50-55%, hypertrophy of the interventricular septum, and mod - erate tricuspid and mitral regurgitation. -Case 2: did not reveal any pathological alteration	-Case 1: Subsequent coronary arteriography showed multivessel coronary artery disease, for which the patient underwent a stent implantation. -Case 2: the patient refused to have further tests	-Case 1: survived -Case 2: recurrence of chest pain occurred when the infusion speed exceeded 50 mL/h with additional cycles.
Sellier-Leclerc et al (21)	‏2013	‏1	A 7-year-old boy	Steroid-dependent idiopathic nephrotic syndrome	Fulminant viral myocarditis	13 months after the first RTX infusion	None	Non- sustained ventricular tachycardia	Cardiac dysfunction	NR	An endomyocardial biopsy was performed which showed an inflammatory infiltrate, vasculitis and myocardial necrosis; PCR assay of the sample was positive for enterovirus; 2 months later the patient successfully underwent heart transplant surgery.
Smith et al (22)	‏2013	1	A 60-year old woman	Lymphoma	Takotsubo cardiomyopa-thy	During infusion of ritux-imab	NR; an echocardiogram 3 days before chemo-therapy was normal	ST-segment elevation in leads V1 to V6	Apical ballooning with hyperdynamic basal segments with an EF of 20-25%	NR	Rituximab was discon-tinued. Discharged home with an angioten-sin-converting enzyme inhibitor and beta-blocker. LVEF was im-proved to 42% at 1 month.
van Sijl et al‏(23)	‏2014	2	70-year old (Case 1) and 76-year old women (Case 2)	Rheumatoid arthritis	Myocardial Infarction	-Case 1: Three months after first treatment -Case 2: One month af-ter the second admin-istration of rituximab	-Case 1: known infero-posterior myocardial infarction post percuta-neous coronary interven-tion. -Case 2: hypertension, hypercholesterolemia, diabetes mellitus or es-tablished coronary heart disease.	-Case 1: ST-elevations suggestive of an anter-olateral myocardial in-farction -Case 2: non-ST elevat-ed myocardial infarction	NR	-Case 1: a thrombus in the LAD for which a stent was placed -Case 2: functional oc-clusion in the diagonal branch of the left anterior descending	-Case 1: One year later, 2 weeks after the second rituximab administration, patient developed cardi-ac arrest. Repeated cor-onary angiogram showed re-stenosis of several coronaries. -Case 2: Had conserva-tive treatment, was dis-charged and doing well at 6-month follow-up.
Mulay et al (35)	‏2015	1	A 69-year-old man	Chronic lymphocytic leukemia	Non-ischemic cardiomy-opathy	During the second and third cycles	History of inferior wall myocardial infarction, hypertension and hyper-lipidemia	NR	Dilated LV and severely reduced LV function with EF of 20% after the sec-ond cycle and 10% after his third cycle. There were no regional wall motion abnormalities.	No significant athero-sclerotic disease noted in the coronary vessel.	The patient received medical treatment for the cardiomyopathy, and required an implantable cardioverter defibrillator for the treatment of symptomatic bradycar-dia.
Ng et al (24)	‏2015	1	A 66-year-old man	Chronic lymphocytic leukaemia	Takotsubo cardiomyopa-thy	Within 40 min of the in-fusion	No previous cardiac his-tory.	Sinus tachycardia; ST segment elevation in leads I, II and V4–V6	Normal LV size with hypokinesis of the ante-rior wall, mild mitral re-gurgitation, aortic regur-gitation and pericardial effusion, but no signs of tamponade.	Non-obstructive distal atheroma with no indica-tion for percutaneous intervention.	Reported no recurrence of his symptoms at fol-low-up.
Cheungpasitporn et al (Our case presentation)	‏2016	1	A 51-year-old man	Membranous nephropathy	Non-ischemic cardiomyopathy	Forty-eight hours after the infusion	No previous cardiac history	New LBBB	Moderately reduced LV systolic function with an EF of 30% with moderate hypokinesis of anteroseptal myocardium.	No significant athero-sclerotic disease noted in the coronary vessels. There was moderate diffuse hypokinesis of LV on ventriculogram.	At 9 month follow-up, despite that he had bet-ter optimization of his medical therapy, his transthoracic echocardi-ogram still showed re-duced LV EF of 31%.

**Table 2:** Summary of cardiovascular adverse effects related to rituximab treatment

**Cardiovascular adverse effects related to rituximab treatment**
Hypotension
Cardiogenic shock
ACS (angina, unstable angina and myocardial infarction)
Arrhythmia (monomorphic ventricular tachycardia, polymorphic ventricular tachycardia, supraventricular tachycardia, trigeminy, bradycardia, atrial fibrillation and nonspecific dysrhythmias or tachycardia)
Reduction in LVEF (may occur within the first few hours after the initial infusion dose or after subsequent doses)
Non-ischemic Cardiomyopathy
Takotsubo’s cardiomyopathy

Interestingly, in a randomized controlled trial of single-dose rituximab as induction in renal transplantation, Tydén et al (27) reported a statistically significant increase in mortality in the rituximab group at 3-year follow-up and 75% of deaths in rituximab-treated recipients were from cardiovascular causes. In a report of 120 patients with follicular and mantle-cell lymphoma treated with rituximab (28), cardiovascular death was reported in 3 of 4 deaths within 12 months. According to the analysis of mortality associated with rituximab use in autoimmune diseases between 2000 and 2013, fatal outcomes were found in 14 patients out of 134 patients (10.4%). While the majority of patients died from infections, 8% died after cardiac events (29). These findings in addition to reported cases of ACS/myocardial infarction associated with the infusion of rituximab (Table 1) raise the concern of its cardiac side effect profile from rituximab since B-lymphocytes, particularly B1a-lymphocytes, were recently found to provide an atheroprotective effect (30). Besides, ACS due to coronary vasospasm following rituximab infusion was also reported (19).

Cardiovascular toxicity in the form of cardiac arrhythmias has been reported in 8% of patients treated with rituximab (2). These include monomorphic ventricular tachycardia, polymorphic ventricular tachycardia, supraventricular tachycardia, trigeminy, bradycardia, atrial fibrillation and nonspecific dysrhythmias or tachycardia (2,18,31-35). It is postulated that the CD 20 antigen also may affect the calcium ion channel (18). The therapeutic action of rituximab may act by cell lysis via complement-dependent cytotoxicity, antibody-dependent cellular cytotoxicity, and apoptosis. The CD20 antigen is present on immune-effector cells and, after cytotoxic-mediated lysis; it could sequester itself in normal tissues of the body, including cardiac myocytes. It is possible that rituximab affects conduction by inhibiting the calcium-ion-channel properties of the CD20 antigen. Inhibition of calcium-ion channels in cardiac myocytes could lead to the formation of early after-depolarization (18).

Kanamori et al (16) reported delayed effect (>2 weeks following rituximab infusion) of reduced LVEF in 3 cases with NHL (Table 1). The prognosis was different in each reported case. Of 3 patients, one had irreversible cardiomyopathy and died with severe heart failure. Partially reversible cardiomyopathy was also recently observed in a case of CLL with cardiomyopathy during treatment with rituximab (24). In contrast to these reported cases, our case presentation provided very important evidence that rituximab can affect cardiac function. Firstly, in previously reported cases of rituximab induced non-ischemic cardiomyopathy; other concurrent chemotherapeutic agents in addition to rituximab were also given. Thus, it was not conclusive that rituximab was the causative agent. In our case presentation, the patient received only rituximab as a single agent for treatment. Secondly, our case had a coronary angiogram which demonstrated clean coronary artery. Thus, we could confirm the diagnosis of non-ischemic cardiomyopathy, unlike previously reported cases (16). Thirdly, our case study showed the new finding that a reduction in cardiac function can occur early within 48 hours. Lastly, this case presentation highlights an importance for obtaining family history prior to rituximab treatment. Our patient’s family history is remarkable for DCM in his brother. Since familial DCM may occur in 20% to 50% of idiopathic DCM cases (36), the patient may have had an occult cardiomyopathy that was symptomatically induced by rituximab infusion.

Although the pathophysiology of rituximab induced non-ischemic cardiomyopathy remains unclear, the finding from Kanamori et al (16) raised an important observation. After rituximab infusion, patients’ cardiac myocytes were noted to have diffuse amounts of reticulin ﬁber along with increased serum transforming growth factor-β levels. The investigators suggested that the transforming growth factor-β levels could have led to increased reticulin ﬁber formation causing a decrease in myocardial contractility leading to non-ischemic cardiomyopathy (16). Also, Takotsubo cardiomyopathy (stress cardiomyopathy), an important type of non-ischemic cardiomyopathy has recently been reported after rituximab infusion (Table 1). In our case presentation, the patient had diffuse hypokinesis which was not a typical finding in Takotsubo cardiomyopathy (37). In addition, most patients with Takotsubo cardiomyopathy recover, unlike our case presentation; LVEF was still significantly reduced at 9-month follow-up.

## Conclusion

In summary, awareness of cardiotoxicity profile of rituximab is important to minimize the risk of treatment-related morbidity/mortality. In addition to ACS and cardiac arrhythmia, non-ischemic cardiomyopathy can be associated with the infusion of rituximab. This condition may occur early within 48 hours. The occurrence of symptoms that could be ascribed to an ACS or heart failure with reduced ejection fraction (HFrEF) should always be taken seriously during the rituximab infusion and carefully investigated. Patients should be aware that this is a rare, albeit serious, complication of treatment with rituximab. Pretreatment echocardiogram may be helpful when considering rituximab treatment in patients who have a family history of cardiomyopathy.

## Authors’ contribution

All authors had access to the data and a role in writing the manuscript.

## Conflicts of interest

Authors declare no conflict of interests.

## Ethical considerations

Ethical issues (including plagiarism, data fabrication, double publication) have been completely observed by authors.

## Funding/Support

None.
